# Incremental validity of the Test of Practical Judgment

**DOI:** 10.1002/alz.090739

**Published:** 2025-01-03

**Authors:** Pranitha Y Premnath, Caroline O Nester, Anjali Krishnan, Nadia Pare, Anna F Wilhelm, Abigail L Zatkalik, David E Warren, Laura Rabin

**Affiliations:** ^1^ Queens College, CUNY, Queens, NY USA; ^2^ The Graduate Center, CUNY, New York, NY USA; ^3^ Brooklyn College, Brooklyn, NY USA; ^4^ Gaylord Specialty Hospital, Wallingford, CT USA; ^5^ University of Nebraska Medical Center, Omaha, NE USA; ^6^ Max Planck School of Cognition, Leipzig Germany; ^7^ Brooklyn College of the City University of New York, Brooklyn, NY USA

## Abstract

**Background:**

Judgment is an aspect of executive functioning that is critical to many aspects of daily functioning, and often affected in older adults with cognitive decline. The Test of Practical Judgement (TOP‐J) evaluates judgment related real‐world issues that may arise in aging populations. The current study investigates the incremental validity of the TOP‐J—i.e., the degree to which the TOP‐J adds unique and meaningful information to a clinical assessment beyond other executive functioning tests which are often used as proxies for judgment.

**Methods:**

Participants (N = 97, *M_age_* = 74.73; 74.2%female) who were classified as cognitively unimpaired (CU), subjective cognitive decline (SCD), or mild cognitive impairment (MCI) completed comprehensive neuropsychological evaluation, which included the Wisconsin Card Sorting Test (64‐card version) and Delis‐Kaplan Executive Function System tests of Verbal Fluency and Trail Making Test, Condition 4. Incremental validity was assessed through hierarchical linear regression analysis and was measured by the addition of the TOP‐J (Forms A, B, a composite of A + B), above these widely used executive functioning tests, with clinical diagnosis (CU, SCD and MCI) as the outcome.

**Results:**

The addition of the TOP‐J A was statistically significant (R^2^ = .299, F(1,90) = 3.95, p = 0.05) and added incremental validity to predict diagnosis (∆R^2^ = .031, p = 0.05). The addition of the TOP‐J forms A + B composite also was significant (R^2^ = .307, F(1,90) = 4.99, p = 0.03, and added incremental validity(∆R^2^ = .038, p = 0.03), while the TOP‐J B trended toward significance (∆R^2^ = .024, p = 0.08).

**Conclusion:**

Our results suggest that including the TOP‐J within neuropsychological evaluations of older adults can enhance diagnostic accuracy and provide clinically valuable information beyond what is derived from traditional executive functioning tests. Considering potential adverse impacts of impaired judgement abilities in the context of neurodegenerative disease, it is of utmost importance to improve the detection of diminished practical judgment skills to increase the protection of vulnerable populations and to prevent potential financial, social, and other areas of exploitation that may occur.

